# Dietary and Lifestyle Patterns are Associated with Heart Rate Variability

**DOI:** 10.3390/jcm9041121

**Published:** 2020-04-14

**Authors:** Elena Reginato, Danila Azzolina, Franco Folino, Romina Valentini, Camilla Bendinelli, Claudia Elena Gafare, Elisa Cainelli, Luca Vedovelli, Sabino Iliceto, Dario Gregori, Giulia Lorenzoni

**Affiliations:** 1 Unit of Biostatistics, Epidemiology and Public Health, Department of Cardiac, Thoracic, Vascular Sciences and Public Health, University of Padova, Via Loredan, 18, 35131 Padova, Italy; elena.reginato@studenti.unipd.it (E.R.); danila.azzolina@uniupo.it (D.A.); luca.vedovelli@unipd.it (L.V.); giulia.lorenzoni@unipd.it (G.L.); 2 Department of Translational Medicine, University of Eastern Piedmont, 28100 Novara, Italy; 3 Cardiology Unit, Department of Cardiac, Thoracic, Vascular Sciences and Public Health, University of Padova, Via Giustiniani, 2, 35128 Padova, Italy; franco.folino@unipd.it (F.F.); sabino.iliceto@unipd.it (S.I.); 4 Dietetics Unit, Department of Medicine, University of Padova, Via Giustiniani, 2, 35128 Padova, Italy; romina.valentini@unipd.it (R.V.); camillabendinelli@gmail.com (C.B.); 5 Department of Nutrition, University of Buenos Aires and Food and Diet Therapy Service, Acute General Hospital Juan A. Fernàndez, C1425 Buenos Aires, Argentina; claudiagafare@gmail.com; 6 Department of Medical Sciences, University of Ferrara, 44100 Ferrara, Italy; cainelli.elisa@gmail.com

**Keywords:** heart rate variability, premature ventricular complexes, supraventricular premature complexes, dietary patterns, lifestyle

## Abstract

Assessment of heart rate variability (HRV) and cardiac ectopic beats is a clinically relevant topic. The present exploratory observational study aimed to inspect the relationships of lifestyle, dietary patterns, and anthropometrics with HRV, premature ventricular complexes (PVCs), and supraventricular premature complexes (SVPCs). A cross-sectional study enrolling subjects undergoing Holter monitoring was performed. Sociodemographic and clinical characteristics, body composition (full-body bio-impedentiometry), dietary patterns (validated food frequency questionnaire and 24 h dietary recall), and quality of life were assessed. Generalized additive models were estimated to evaluate the relationships between outcomes of interest and variables collected. The study enrolled 121 consecutive patients undergoing 24 h Holter monitoring. Upon univariable analysis, HRV was found to have an inverse association with mass of body fat (MBF) (*p*-value 0.015), while doing physical activity was associated with a significantly higher HRV (*p*-value 0.036). Upon multivariable analysis, fruit consumption in the 24 h dietary recall was found to be directly associated with HRV (*p*-value 0.044). The present findings might be useful for improving the management of patients attending cardiac rhythm labs, and to tailor ad hoc prevention strategies (modification of lifestyle and eating habits) based on Holter parameters.

## 1. Introduction

Globally, the leading cause of death is cardiovascular diseases (CVDs); their prevalence is incessantly progressing in both developed and developing nations [1]. Although factors such as age, sex, and family history are considered crucial, some established factors are modifiable: hypertension, use of tobacco, diabetes mellitus, physical inactivity, unhealthy diet, cholesterol and lipids, and stress, among others [2]. Risk factors are often observed in clusters, so that even if just one risk factor such as hypertension is detected in a person, a search for coexisting risk factors such as smoking, central adiposity, hyperlipidemia, and diabetes mellitus becomes obligatory, because these risk factors occurring together can increase the risk of CVDs in a multiplicative rather than in an additive manner [3]. Though the prognosis of cardiovascular disease has improved because of better medical care, research on prognostic factors remains central. Heart rate variability (HRV) assessment is a clinically relevant topic, since even small alterations can predict severe, life-threatening, cardiac arrhythmias and cardiovascular events [4]. In recent decades, HRV has been extensively studied as a surrogate marker of autonomic function. Studies have been conducted both in healthy subjects and in those with underlying conditions affecting the cardiovascular system (e.g., previous myocardial infarction, congestive heart failure) [5]. Abnormal HRV (reduced) has been reported for patients suffering from myocardial infarction and diabetic neuropathy [5]. Although it has been shown that reduced HRV is a significant predictor of mortality and cardiovascular events, especially in patients with underlying cardiovascular diseases [6], it is still not entirely clear if reduced HRV represents the result of such diseases or the factor affecting the onset of such conditions.

Together with HRV, the role of cardiac ectopic beats (supraventricular premature complexes (SVPCs) and premature ventricular complexes (PVCs)) has been widely studied in the literature. More attention has been paid to PVCs, since PVCs is considered generally harmless but possibly a predictor of severe malignant cardiac arrhythmias (e.g., ventricular fibrillation).

Given the prognostic relevance of HRV and PVCs, it is crucial to understand the lifestyle factors and mechanisms associated with such parameters. In recent years, a role of anthropometrics, dietary patterns, and lifestyle in affecting cardiac ectopic beats and HRV has been suggested; however, evidence remains limited and unclear. In addition, there is a growing body of literature about the central role of some nutrients in the prevention of cardiac arrhythmias [7], especially fatal arrhythmias (ventricular arrhythmias), but little evidence is available regarding the protective effects of dietary patterns and lifestyle on PVCs and HRV.

Results from randomized controlled trials suggest that the supplementation of omega-3 polyunsaturated fatty acids (PUFA) may reduce the number of PVCs per day [8], also decreasing their severity [9]. Moreover, omega-3 PUFAs have also been hypothesized to be linked to HRV, supporting their protective role in subjects at high risk for arrhythmic events [10] and sudden cardiac death (SCD) [11]. Together with substances that may play a role in preventing PVCs, some food contents may act as triggers. Some studies have suggested that alcohol and caffeine consumption may be linked to cardiac performance. Evidence indicates the existence of an association between different doses of alcohol consumption with HRV [12] and cardiac ectopic beats [13], while the role of caffeine is still disputed.

Not only the type of food intake, but also meal frequency has been suggested to affect cardiovascular risk profile; it has been suggested that having late dinner may be a proxy for atrial arrhythmias [14], but evidence in the field is still scarce.

Together with dietary patterns, anthropometrics and lifestyle have also been hypothesized to affect HRV and cardiac ectopic beats. Physical activity has been demonstrated to be a relevant factor in improving HRV [15]. Regarding anthropometrics, obesity and being overweight represent a predictor of cardiac functions’ impairment. A higher number of PVCs and reduced HRV have been documented in obese subjects [16]. Additionally, a recent research study identified the waist-to-hip ratio, a marker of visceral adiposity, to be associated with HRV [17].

Although studies have suggested the existence of an association between lifestyle, HRV, and cardiac ectopic beats, the underlying mechanisms are not yet clear and evidence is limited and controversial. More specifically, data regarding PVCs and HRV are lacking, despite the prognostic relevance of PVCs and HRV in clinical practice.

The present exploratory observational study aimed to inspect relationships of dietary patterns, anthropometrics and lifestyle, with HRV, PVCs, and SVPCs. In particular, it aimed at analyzing if any difference exists in the role played by such factors on outcomes of interest in patients undergoing Holter monitoring.

## 2. Materials and Methods

This study was a cross-sectional exploratory investigation conducted at the Department of Cardiac, Thoracic, Vascular Sciences and Public Health of the University of Padova, Azienda Ospedaliera di Padova. It enrolled patients referred to the outpatient clinic for 24 h Holter monitoring, from May to July 2016.

To be enrolled in the study, patients were required to meet the following criteria: older than 18 years of age; ability to speak and understand the Italian language; and absence of cognitive impairment. Patients were asked to give written informed consent to participate in the study. The study was approved by the competent Institutional Review Board.

### 2.1. Holter Monitor

Patients were invited to wear a Holter monitor for 24 h and were provided with a diary in which to report activities and symptoms perceived during the 24 h Holter monitoring, with the final aim of finding potential associations between symptoms felt, reported in the diary, and modifications of heart activity documented by the Holter monitor.

Holter monitor data considered in this study were minimum and maximum heart rate, PVCs, SVPCs, and HRV (standard deviation of the N-N (SDNN) intervals in ms). For this study, PVCs, SVPCs, and HRV were considered as outcomes of interest. 

### 2.2. Demographic Characteristics, Lifestyle, and Clinical Assessment

Demographic characteristics, lifestyle habits, and clinical history were collected through a purpose-designed questionnaire, administered by an interviewer, and available medical records.

Collected demographic characteristics were age, sex, educational level (categorized as “low”, “medium”, and “high”, corresponding to primary school, high school, and bachelor’s/master’s degree, respectively), and employment situation. Investigated lifestyle habits were tobacco smoking, physical activity, hours of sleep, and leisure activities. Clinical history focused on concomitant medications and comorbidities.

### 2.3. Health-Related Quality of Life (HRQoL)

Health-related quality of life was assessed using the EuroQol-5D 3 level version (EQ-5D-3L). EQ-5D is a self-administered questionnaire which consists of two parts: the EQ-5D descriptive system and the EQ visual analogue scale (EQ-VAS). EQ-5D focuses on five dimensions: mobility, self-care, usual activities, pain/discomfort, and anxiety/depression. Three levels are available for each dimension: “no problems”, “some problems”, and “severe or extreme problems”. Participants were asked to tick the option preferred for each dimension.

The EQ-VAS consists on a vertical visual analogue scale ranging from “best imaginable health state” to “worst imaginable health state”, corresponding respectively to 100 and 0 scores. Patients were asked to indicate in the scale the numeric value better describing their quality of life.

Psychometric properties, including reliability and construct validity, have been already assessed for the Italian version of EuroQol-5D [18].

### 2.4. Anthropometric Data

Patients underwent anthropometric assessment by trained dietitians. Weight, height, hip, waist and arm circumference were measured and body mass index (BMI) was computed dividing weight (in kilograms) by height squared (in meters). Tricep skinfold was measured using a Harpenden skinfold caliper.

Participants underwent body composition analysis using a D-1000-3 Full Body Analyzer (Heteren, Netherlands, Rice Lake^®^). The bioelectrical impedance analysis (BIA) measures body composition through the impedance of the human body, providing values of mass of body fat (MBF), lean body mass (LBM), total body water (TBW), intracellular water (ICW), body mass index (BMI), percentage of body fat (PBF), and segmental lean body mass of body parts (trunk, arms, and legs).

Patients with implantation of metallic material (e.g., stents) and/or of devices making electric signals (e.g., artificial heart), connection to electronic equipment with good conductivity, taking contraceptives, suspected to be pregnant, or susceptible to a small amount of electrical stimulation were excluded from the BIA assessment, according to the safety rules for the D-1000-3 Full Body Analyzer (Heteren, Netherlands, Rice Lake^®^).

### 2.5. Food Frequency Questionnaire (FFQ) and 24 h Dietary Recall

Patients’ eating habits were assessed using a brief version of the Turconi Food Frequency Questionnaire (FFQ). For the study, only two sections of the FFQ were administered, the one about the frequency of food consumption, and the one on eating habits. The first one consists of questions about the frequency of food consumption (daily, weekly, etc.) of food products from different food groups, including beverages. The section on eating habits consists of 14 questions that focus mainly on meal frequency, drinks, and food consumption during meals and outside mealtimes. A score is assigned to each question. The score ranged from 1 (less healthy habits) to 4 (more healthy habits). The total possible score of this second section is 56. Higher scores mean better eating habits. The questionnaire was previous validated in a sample of Italian adults [19].

The day after the Holter monitoring, 24 h dietary recall was collected, asking about food consumption during the 24 h of Holter monitoring. Food reported by patients at mealtimes was categorized using “What we Eat in America” (WWEIA) [20] food categories, an instrument proposed by the United States Department of Agriculture (USDA) to investigate food and beverage intake. The classification consists of three levels based on grouping similar foods and beverages based on nutrient content, without disaggregation into ingredients.

The choice of a U.S. classification tool was motivated by the fact that it was considered more suitable for the purpose of our study compared to that proposed from the European Food Safety Authority (EFSA), since that of EFSA is generally employed in total diet studies [21].

### 2.6. Analysis of Precision

Being an observational prospective study, it was designed to guarantee attainment of a desired target of precision in estimates. Relative precision was computed for mean difference in the number of meals (indicated below as X) during the 24 h dietary recall, between patients with and without cardiac ectopic beats (registered by Holter monitor), considering

- the target difference in the number of meals between groups, defined as ${\bar{X}}_{1}-{\bar{X}}_{2}$ = 2;
- the pooled standard error of the difference between groups, defined as S=2.5;
- significance level set at 0.05.

Parameters above were derived from pre-existing clinical records. After having evaluated the patients’ distribution, an overall sample of 100 subjects, not balanced by the presence/absence of cardiac ectopic beats, was considered reasonable for the study. Under such assumptions, a relative precision of 4.43% of point estimate was expected.

### 2.7. Statistical Analysis

Categorical data were reported as relative and absolute frequencies, and continuous data as median and Quartiles I and III. Wilcoxon–Kruskal–Wallis tests were performed for continuous variables and Pearson chi-square tests for categorical ones.

For multiple-choice variables, multiple marginal independence (MMI) was tested, using nonparametric bootstrap resamples under the null hypothesis of independent sampling. A modified Pearson statistic was calculated for each resample (*n* = 2000).

PVCs and SVPCs were treated as semi-continuous data, since they presented a combination of both a point-mass at zero and a positively skewed distribution (as clearly shown in Figures S1 and S2, Supplementary Material). Such data are unsuitable for analysis using positively skewed distributions since distributions that are unbounded are likely to result in a poor fit [22]. To account for such a distribution, a generalized additive model, considering a zero adjusted gamma distribution (ZAGA) [23] for dependent variables, was estimated to evaluate the effect of independent variables on PVCs and SVPCs. More specifically, for both models, the probability that an outcome was non-zero was modeled via logistic regression. The distribution of the non-zero outcomes was modeled via gamma regression with a log-link for ZAGA. This type of non-negative data distribution is commonly found in data from many research fields, and, recently, this approach has also been applied in nutrition and dietary research [24].

HRV was modeled using a generalized additive model with gamma distribution considering the skewness of dependent variable (Figure S3, Supplementary Material).

Evaluation of the goodness of fit of the multivariable estimated models was performed using graphical inspection of theoretical and estimated residuals’ quantiles and via a Kernel density estimation of residual distribution.

*P*-values related to the effects of multiple testing were adjusted using the Benjamini and Hochberg procedure.

Computations were performed using R System 3.3.1 software [25] with rms, gamlss, and MRCV packages.

## 3. Results

One hundred and twenty-one consecutive patients attending the cardiac rhythm lab for 24 h Holter monitoring were enrolled in the study. The effects of single variables on PVCs, SVPCs, and HRV are reported in Table 1.

### 3.1. Demographic and Lifestyle

Ninety-five patients had at least 1 PVCs (reported by the 24 h Holter monitoring), with a median of 78 PVCs (13–746, Quartiles I-III). Sociodemographic characteristics and lifestyle in presence/absence of PVCs are reported in Table 1. Subjects with PVCs were significantly older (median age of 67 vs. 44, p 0.006) compared to those who did not suffer from it (Table 1).

Subjects’ characteristics according to presence/absence of SVPCs are reported in Supplementary material (Table S1). Briefly, 109 subjects out of 121 presented with at least one SVPCs (median of 39, 9-224 Quartiles I-III). No statistical differences were found in sociodemographic characteristics and lifestyle in the presence/absence of SVPCs.

Regarding Holter parameters, no significant differences were reported, except for the number of SVPCs (PVCs subjects had a higher number of SVPCs compared to non-PVCs subjects, *p*-value < 0.001) (Table 2).

Regarding the univariable analysis (Table 3), data showed that doing physical activity was associated with a significantly higher HRV (*p*-value 0.036), but not with PVCs and SVPCs. Higher age and the presence of cardiovascular comorbidities were shown to result in a significantly higher likelihood of SVPCs (both *p*-value < 0.001).

In the multivariable analysis (Table 4), age appeared to be associated with PVCs (*p*-value 0.005) and SVPCs appeared to be associated with age and sex (respectively, *p*-value < 0.001 and *p*-value 0.004).

Univariable and multivariable analyses for PVCs, SVPCs, and HRV are reported in Table 3 and Table 4, respectively.

### 3.2. Anthropometrics

No statistical differences in presence/absence of PVCs and SVPCs were found for anthropometrics (Table 1 and Table S1, respectively).

Regarding results of univariable analyses (Table 3), HRV was found to be in a direct (but nonsignificant, *p*-value 0.076) association with LBM. Conversely, it was found to be in an inverse association with MBF (*p*-value 0.015). Both LBM and MBF appeared to not affect SVPCs, while MBF appeared to be directly associated with PVCs (p-value 0.015). No statistical significance of BMI on outcomes was observed, whereas waist circumference was found to be directly associated with higher likelihood of both PVCs and SVPCs (*p*-value 0.024 and <0.001, respectively). In the multivariable analysis (Table 4), higher BMI was associated with lower HRV (although not significantly, *p*-value 0.083).

### 3.3. Dietary Pattern

No statistical differences in presence/absence of PVCs and SVPCs were found for dietary pattern (Table 1 and Table S1, respectively). Regarding 24 h dietary recall, no significant effects of single variables were observed, with only a few exceptions (e.g., consumption of condiments and sauces during the 24 h of Holter monitoring appeared to result in a higher risk of SVPCs; *p*-value 0.063, barely significant). Similar results were obtained from the analysis of the frequency of food consumption (assessed through the Turconi FFQ) (Table 3).

Upon multivariable analysis (Table 4), fruit consumption during the 24 h dietary recall was found to be directly associated with HRV (*p*-value 0.044). Regarding PVCs, results showed that it was significantly directly associated with higher intake of grain-based products (*p*-value 0.001) and consumption of snacks and sugars (*p*-value 0.063 and 0.013, respectively), whereas fruit intake was found to be significantly and inversely associated with PVCs (*p*-value 0.024). Consumption of condiments and sauces raised the likelihood of SVPCs (*p*-value 0.01), while protein food consumption was significantly and inversely proportional to SVPCs (*p*-value < 0.001).

## 4. Discussion

Results of our exploratory observational study suggest that lifestyle, eating habits, and body adiposity are significantly associated with cardiac ectopic beats and HRV.

Consistently with previous findings [26], the results of our study pointed out the close relationship between body composition and the outcomes of interest. The univariable analysis showed that waist circumference was significantly associated with both PVCs and SVPCs, in accordance with the growing body of literature which supports the relevant role played by the waist and hip circumference in cardiac risk predictive models [27]. Conversely to what is documented in the literature [28], our study showed no significant results regarding the effect of BMI on cardiac ectopic beats. In contrast from BMI, MBF resulted to be significantly directly associated to PVCs. This is consistent with evidence reported in the literature; a recent review documented the role of adipose tissue on cardiac arrhythmias, and in particular on ventricular rhythm disorders [29].

Regarding food intake, we found out that higher fruit consumption had a significant impact on the improvement of HRV and in reduced occurrence of PVCs. Despite the limited availability of evidence regarding potential associations between fruit intake and HRV and ectopic beats, the beneficial role of fruit in improving cardiac autonomic function has been documented [30].

Upon multivariable analysis, age was found to be associated with SVPCs and PVCs, but not with HRV; this finding is only partially consistent with literature in the field [31]. Regarding dietary patterns, our study showed a significant association between higher intake of snacks and sweets, sugars, and grain products with PVCs. To our knowledge, this is the first study in which the consumption of such food products has been investigated regarding its linkage to cardiac ectopic beats and HRV. Despite the lack of evidence regarding cardiac rhythm, studies have reported the potential risk of refined carbohydrates and simple sugars in facilitating weight gain [32], thus increasing cardiovascular risk. To reduce such risk, evidence suggests limiting the consumption of refined grains and to prefer low energy carbohydrates, such as whole grain cereals, fruit, vegetables, and legumes.

Regarding the consumption of protein, the present study suggested that protein intake was inversely associated with SVPCs. However, no specifications were made as to protein type (animal vs. plant). In the literature, limited evidence is available regarding the association between protein food and cardiac electrical activity, since previous studies have concentrated mainly on fish intake and cardiac rhythm, not on another type of protein food. Results of such studies suggested a beneficial role of omega-3 PUFA in fish against cardiac arrhythmias [33].

Together with dietary intake, lifestyle habits also appeared to be significantly associated with outcomes of interest. Consistently with recent publications, our study showed that physical activity was significantly associated with higher HRV (by reducing sympathetic activation [15]). Sleep hours also resulted in a significant relationship with HRV and SVPCs; sleep hours had a direct relationship with HRV and an inverse relationship with SVPCs. The effect on HRV is physiological, since it is well known that longer sleep time results in higher HRV, while poor sleeping or sleep deprivation can adversely affect HRV [34]. Conversely, no significant effects of sleep hours on PVCs were observed, whereas recent research has pointed out higher PVCs as a consequence of sleep disruption in hospitalized patients [35].

Some differences between this study and previous findings may be due to different data collection methods, especially regarding the 24 h recall. Generally, previous studies employed only FFQ to estimate the effect of food consumption on cardiac rhythm.

Despite the fact that this was an exploratory study and significant results should be researched further by independent labs in a more targeted research design, these findings provide new insights to improve the management of patients attending cardiac rhythm labs.

Holter monitoring is a clinical investigation that helps medical doctors to assess cardiac function, especially if a standard electrocardiogram does not give enough information about heart electrical activity. This instrumental examination generally falls under a set of clinical tests intended to be used for differential diagnosis. As a result, data from the Holter monitor are often underused, especially in the research context. Conversely, the findings of the present study provide new insights into the usefulness of such clinical examination. If lifestyle and dietary patterns influence the onset (and/or the severity) of cardiac rhythm disorders, clearly, Holter monitor parameters may support clinicians in the development of ad hoc strategies (modification of lifestyle and eating habits) based on Holter parameters to improve cardiac electrical function, reducing the risk of more severe, life-threating, cardiac arrhythmias.

## 5. Conclusions

Results of our study suggested that lifestyle, eating habits, and anthropometrics were significantly associated with cardiac ectopic beats and HRV.

To our knowledge, this is the first study to point out the association of such heterogeneous factors, from food intake to lifestyle habits and anthropometrics, on cardiac rhythm outcomes, providing new insight to the topic. Given the clinical relevance of HRV, SVPCs, and PVCs on morbidity and mortality, it may be useful to implement research in this field to improve clinical management (including both drugs and lifestyles prescriptions) of patients with such cardiac rhythm disorders, that, even small, may be relevant prognostic factors for more severe cardiac arrhythmias, especially in subjects with underlying cardiovascular diseases.

## Figures and Tables

**Table 1:** Descriptive statistics according to presence/absence of PVCs. Data are percentages (absolute numbers) for categorical variables, and Quartile I/median/Quartile III for continuous variables.

	Premature Ventricular Complexes: No (*n* = 26)	Premature Ventricular Complexes: Yes (*n* = 95)	Combined (*n* = 121)	*p*-Value
Sociodemographic Characteristics and Lifestyle				
Age	29.75/44.00/66.75	50.50/67.00/77.00	47.00/66.00/75.00	0.006
Sex: Male	38% (10)	45% (43)	44% (53)	0.756
Female	62% (16)	55% (52)	56% (68)	
Educational level: Low	38% (10)	47% (45)	45% (55)	0.723
Medium	42% (11)	29% (28)	32% (39)	
High	19% (5)	23% (22)	22% (27)	
Employment: No	54% (14)	67% (64)	64% (78)	0.496
Yes	46% (12)	33% (31)	36% (43)	
Cardiovascular comorbidities: No	69% (18)	35% (33)	42% (51)	0.06
Yes	31% (8)	65% (62)	58% (70)	
Concomitant medications. Ace inhibitors: Yes	23% (6)	41% (39)	37% (45)	0.496
Diuretics: Yes	19% (5)	26% (25)	25% (30)	
Potassium supplements: Yes	0% (0)	2% (2)	2% (2)	
Flecainide: Yes	4% (1)	5% (5)	5% (6)	
Class I antiarrhythmic agents: Yes	4% (1)	0% (0)	1% (1)	
Class III antiarrhythmic agents: Yes	0% (0)	3% (3)	2% (3)	
Digoxin: Yes	0% (0)	2% (2)	2% (2)	
Nitrates: Yes	0% (0)	3% (3)	2% (3)	
Calcium channel blockers: Yes	8% (2)	17% (16)	15% (18)	
Beta blockers: Yes	19% (5)	39% (37)	35% (42)	
Vasodilators: Yes	0% (0)	1% (1)	1% (1)	
Platelets aggregation inhibitors: Yes	19% (5)	34% (32)	31% (37)	
Anticoagulants: Yes	8% (2)	15% (14)	13% (16)	
Cholesterol lowering medications: Yes	19% (5)	32% (30)	29% (35)	
Insulin: Yes	0% (0)	4% (4)	3% (4)	
Oral hypoglycemic agents: Yes	4% (1)	9% (9)	8% (10)	
Smoking habit: No	62% (16)	58% (55)	59% (71)	0.285
Past smoker	12% (3)	33% (31)	28% (34)	
Current smoker	27% (7)	9% (9)	13% (16)	
Cigarettes number (per day)	7.00/10.00/20.00	5.00/ 8.00/15.00	4.75/ 9.00/20.00	0.756
Physical activity: No	50% (13)	61% (58)	59% (71)	0.583
Yes	50% (13)	39% (37)	41% (50)	
Physical activity: number of weekly training sessions	3/3/5	2/3/3	2/3/4	0.411
Sleep hours	6.0/6.5/7.0	5.0/6.5/7.0	5.0/6.5/7.0	0.883
EQ5D VAS	60.00/70.00/80.00	51.00/70.00/80.00	57.75/70.00/80.00	0.938
Anthropometrics and BIA				
Lean body mass	43.50/45.70/48.07	44.15/47.05/59.17	43.85/46.20/52.92	0.496
Total body water	31.35/32.90/34.60	31.80/33.90/42.57	31.57/33.25/38.10	0.496
Extracellular water	12.10/12.90/14.05	12.77/13.95/17.22	12.57/13.75/15.52	0.375
Mass of body fat	10.87/18.50/24.10	16.52/20.25/21.95	14.85/19.75/22.37	0.748
Percent of body fat	17.70/30.75/34.60	22.15/29.35/31.80	21.60/29.65/33.85	0.951
BMI	21.75/23.58/27.62	22.52/24.77/27.59	22.28/24.66/27.67	0.723
Skinfold thickness	11.57/18.43/26.08	11.67/18.30/23.17	11.57/18.30/23.91	0.82
Waist	81.12/ 90.00/ 98.75	87.00/ 95.00/103.00	86.00/ 95.00/103.00	0.496
Hip	94.0/100.5/106.0	98.0/103.0/108.0	96.0/102.0/107.5	0.583
Food Frequency Questionnaire				
Score of eating habits section	39.50/44.00/48.75	42.00/46.00/48.50	42.00/45.00/49.00	0.602
Daily milk/yogurt: No	35% (9)	27% (26)	29% (35)	0.723
Yes	65% (17)	73% (69)	71% (86)	
If yes, how many? 1–2	100% (17)	94% (65)	95% (82)	0.583
3–4	0% (0)	6% (4)	5% (4)	
If no, weekly milk/yogurt: at least once a week	22% (2)	35% (9)	31% (11)	0.735
Less than once a week	78% (7)	65% (17)	69% (24)	
Daily grains: No	27% (7)	19% (18)	21% (25)	0.639
Yes	73% (19)	81% (77)	79% (96)	
If yes, how many? 1–2	100% (19)	99% (76)	99% (95)	0.789
3–4	0% (0)	1% (1)	1% (1)	
If no, weekly grains: 1–2	14% (1)	25% (5)	22% (6)	0.496
3–4	86% (6)	45% (9)	56% (15)	
>4	0% (0)	30% (6)	22% (6)	
Daily fruits and vegetables: No	31% (8)	12% (11)	16% (19)	0.285
Yes	69% (18)	88% (84)	84% (102)	
If yes, how many? 1–2	61% (11)	76% (64)	74% (75)	0.496
3–4	39% (7)	20% (17)	24% (24)	
>4	0% (0)	4% (3)	3% (3)	
Weekly servings of meat: at least once a week	65% (17)	76% (72)	74% (89)	
At least once a day	15% (4)	11% (10)	12% (14)	
Less than once a week	19% (5)	14% (13)	15% (18)	
Weekly servings of fish: 1–2	46% (12)	59% (56)	56% (68)	0.781
3–4	19% (5)	13% (12)	14% (17)	
>4	0% (0)	1% (1)	1% (1)	
every 10–15 days	15% (4)	17% (16)	17% (20)	
never	19% (5)	11% (10)	12% (15)	
Weekly servings of eggs: at least once a week	73% (19)	82% (78)	80% (97)	
Less than once a week	27% (7)	18% (17)	20% (24)	
Weekly servings of cheese: 1–2	35% (9)	42% (40)	40% (49)	0.883
3–4	23% (6)	25% (24)	25% (30)	
>4	19% (5)	19% (18)	19% (23)	
every 10–15 days	12% (3)	5% (5)	7% (8)	
never	12% (3)	8% (8)	9% (11)	
Weekly servings of cured meat: 1–2	15% (4)	48% (46)	41% (50)	0.375
3–4	19% (5)	9% (9)	12% (14)	
>4	12% (3)	6% (6)	7% (9)	
every 10–15 days	31% (8)	21% (20)	23% (28)	
never	23% (6)	15% (14)	17% (20)	
Weekly servings of legumes: 1–2	31% (8)	52% (49)	47% (57)	0.411
3–4	31% (8)	17% (16)	20% (24)	
>4	8% (2)	2% (2)	3% (4)	
every 10-15 days	12% (3)	20% (19)	18% (22)	
never	19% (5)	9% (9)	12% (14)	
Weekly servings of cakes: 1–2	11% (2)	29% (25)	26% (27)	0.411
3–4	16% (3)	9% (8)	10% (11)	
1 per day	58% (11)	28% (24)	33% (35)	
2 per day	11% (2)	12% (10)	11% (12)	
every 10–15 days	5% (1)	14% (12)	12% (13)	
never	0% (0)	8% (7)	7% (7)	
Weekly servings of French fries: 1–2	12% (3)	9% (9)	10% (12)	0.95
3–4	0% (0)	1% (1)	1% (1)	
every 10–15 days	27% (7)	24% (23)	25% (30)	
never	62% (16)	65% (62)	64% (78)	
How often do you eat fast food per week? (weekly) 1–2	0% (0)	2% (2)	2% (2)	0.496
every 10–15 days	12% (3)	3% (3)	5% (6)	
never	88% (23)	95% (90)	93% (113)	
How often do you eat at a pizzeria? (weekly) 1–2	32% (8)	23% (22)	25% (30)	0.411
3–4	48% (12)	33% (31)	36% (43)	
every 10-15 days	20% (5)	44% (42)	39% (47)	
Do you drink wine: No	62% (16)	42% (40)	46% (56)	0.411
Yes	38% (10)	58% (55)	54% (65)	
If yes, how many? (weekly) 1–2	10% (1)	16% (9)	15% (10)	0.639
3–4	20% (2)	11% (6)	12% (8)	
every 10–15 days	40% (4)	20% (11)	23% (15)	
every day	30% (3)	53% (29)	49% (32)	
Do you drink beer? No	58% (15)	72% (68)	69% (83)	0.496
Yes	42% (11)	28% (27)	31% (38)	
If yes, how many? (weekly): 1–2	45% (5)	41% (11)	42% (16)	0.938
3–4	9% (1)	4% (1)	5% (2)	
every 10–15 days	36% (4)	44% (12)	42% (16)	
every day	9% (1)	11% (3)	11% (4)	
Do you drink other alcoholic beverages? No	80% (20)	79% (74)	79% (94)	0.938
Yes	20% (5)	21% (20)	21% (25)	
If yes, how many? (weekly): 1–2	20% (1)	50% (10)	44% (11)	0.723
3–4	0% (0)	5% (1)	4% (1)	
every 10–15 days	60% (3)	40% (8)	44% (11)	
every day	20% (1)	5% (1)	8% (2)	
Do you drink spirits? No	96% (25)	96% (91)	96% (116)	0.95
Yes	4% (1)	4% (4)	4% (5)	
24 h Dietary Recall				
Number of meals in the 24 h recall	3/4/5	3/4/5	3/4/5	0.583
Alcoholic beverages	1.00/2.00/2.75	0.00/1.00/2.00	0.00/1.00/2.00	0.561
Non-alcoholic beverages	0/0/1	0/1/1	0/0/1	0.411
Condiments and sauces	0/1/1	0/1/1	0/1/1	0.781
Fats and oils	0.0/0.5/1.0	0.0/1.0/2.0	0.0/1.0/2.0	0.496
Fruit	0/1/2	1/2/2	1/1/2	0.375
Grain products	0/1/2	0/1/1	0/1/2	0.82
Milk and dairy	0/1/2	0/1/1	0/1/2	0.82
Mixed dishes	0.0/0.5/1.0	0.0/0.0/1.0	0.0/0.0/1.0	0.288
Potatoes: No	88% (23)	95% (90)	93% (113)	0.561
Yes	12% (3)	5% (5)	7% (8)	
Protein food	0/1/1	0/1/1	0/1/1	0.883
Snacks and sweets	0/1/2	0/1/1	0/1/2	0.496
Sugars	0.00/1.00/1.75	0.00/1.00/2.00	0.00/1.00/2.00	0.781
Vegetables	0/1/2	0/1/2	0/1/2	0.938
Water	0/2/2	0/1/2	0/1/2	0.496

**Table 2:** Holter monitor variables according to presence/absence of PVCs. Data are Quartile I/median/Quartile III.

	Premature Ventricular Complexes: No (*n* = 26)	Premature Ventricular Complexes: Yes (*n* = 95)	Combined (*n* = 121)	*p*-Value
Heart beats	85404.0/100830.0/112876.0	84779.0/94147.0/105342.5	85075.5/95447.5/106228.8	0.442
Mean heart rate	67.0/72.0/78.0	61.5/69.0/76.0	62.0/70.0/77.0	0.442
Heart rate: Minimum	45.00/48.00/54.00	s41.50/48.00/54.50	42.75/48.00/54.25	0.959
Heart rate: Maximum	102.0/115.0/121.0	97.5/116.0/133.0	98.0/116.0/132.0	0.959
Heart rate variability	115.0/139.9/174.0	103.1/135.3/173.2	103.7/137.0/174.6	0.959
Supraventricular Premature complexes	0.00/7.50/38.75	13.50/54.00/326.00	9.00/39.00/224.00	<0.001

**Table 3:** Results of univariable analyses for premature ventricular complexes, supraventricular premature complexes, and heart rate variability. Exp estimate represents E[(Y+1); X]/ E [Y; X] indicating the relative variation in the expected number of arrhythmic events consequently to a unit increase of each considered X variable.

	Premature Ventricular Complexes			Supraventricular Premature Complexes			Heart Rate Variability		
	Estimate (Standard Error)	Exp. Estimate	*p*-Value	Estimate (Standard Error)	Exp. Estimate	*p*-Value	Estimate (Standard Error)	Exp. Estimate	*p*-Value
Sociodemographic characteristics and lifestyle									
Age	0.021 (0.011)	1.021	0.112	0.06 (0.009)	1.062	<0.001	−0.002 (0.002)	0.998	0.253
Sex: female vs. male	−0.618 (0.386)	0.539	0.336	−0.3 (0.342)	0.741	0.462	−0.053 (0.072)	0.948	0.462
Educational level: medium vs. low	−0.108 (0.452)	0.898	0.82	−0.088 (0.386)	0.916	0.82	0.057 (0.082)	1.059	0.82
Educational level: high vs. low	−0.682 (0.488)	0.506	0.248	−1.038 (0.438)	0.354	0.057	−0.023 (0.094)	0.977	0.803
Employment: Yes vs. No	−1.567 (0.398)	0.209	<0.001	−0.226 (0.354)	0.798	0.524	0.059 (0.075)	1.061	0.524
Cardiovascular Comorbidities: Yes vs. No	0.527 (0.405)	1.694	0.294	1.723 (0.321)	5.604	<0.001	−0.016 (0.072)	0.985	0.831
Tobacco smoking: Past smoker vs. Never smoked	0.46 (0.421)	1.585	0.414	−0.576 (0.382)	0.562	0.402	0.043 (0.081)	1.044	0.601
Tobacco smoking: Current smoker vs. Never smoker	−0.7 (0.673)	0.496	0.45	−1.197 (0.515)	0.302	0.066	−0.067 (0.106)	0.935	0.53
How many cigarettes/day	−0.048 (0.053)	0.953	0.609	−0.033 (0.039)	0.967	0.609	−0.004 (0.009)	0.996	0.657
Physical activity: Yes vs. No	0.258 (0.397)	1.294	0.517	−0.315 (0.347)	0.73	0.517	0.181 (0.07)	1.199	0.036
specify frequency per week	0.161 (0.16)	1.175	0.477	−0.043 (0.157)	0.958	0.786	0.032 (0.024)	1.032	0.477
Hours slept during the night	−0.084 (0.132)	0.92	0.527	−0.208 (0.113)	0.812	0.115	0.042 (0.024)	1.043	0.115
EQ5D VAS	−0.006 (0.009)	0.994	0.735	−0.003 (0.008)	0.997	0.735	0.002 (0.002)	1.002	0.735
Anthropometrics and BIA									
Lean body mass	0.052 (0.026)	1.054	0.076	0.003 (0.028)	1.003	0.923	0.012 (0.005)	1.012	0.076
Total body water	0.073 (0.036)	1.076	0.076	0.004 (0.039)	1.004	0.925	0.016 (0.007)	1.016	0.076
Extracellular water	0.186 (0.085)	1.205	0.098	0.062 (0.092)	1.064	0.505	0.032 (0.017)	1.033	0.098
Mass of body fat	0.142 (0.053)	1.153	0.015	0.075 (0.046)	1.078	0.113	−0.023 (0.008)	0.977	0.015
Percentage of body fat	0.035 (0.047)	1.035	0.468	0.045 (0.041)	1.046	0.42	−0.024 (0.007)	0.977	0.003
BMI	0.063 (0.05)	1.065	0.315	0.008 (0.043)	1.008	0.85	−0.016 (0.009)	0.984	0.216
Skinfold thickness	−0.007 (0.023)	0.993	0.754	−0.011 (0.021)	0.989	0.754	−0.007 (0.004)	0.993	0.333
Waist circumference	0.038 (0.015)	1.038	0.024	0.051 (0.013)	1.052	<0.001	−0.004 (0.003)	0.996	0.203
Hip circumference	0.025 (0.018)	1.025	0.449	0.014 (0.016)	1.015	0.449	−0.002 (0.003)	0.998	0.449
Food Frequency Questionnaire									
Score of eating habits section	−0.016 (0.037)	0.984	0.67	0.023 (0.031)	1.023	0.67	0.005 (0.006)	1.005	0.67
Daily milk/yogurt: Yes vs. No	0.146 (0.434)	1.157	0.738	−0.254 (0.38)	0.776	0.738	−0.068 (0.077)	0.934	0.738
If yes. daily servings: 3–4 vs. 1–2	−2.007 (0.956)	0.134	0.117	0.208 (0.873)	1.231	0.882	0.03 (0.203)	1.031	0.882
If no. weekly servings: less than once a week vs. at least once a week	−0.327 (0.788)	0.721	0.681	−1.372 (0.745)	0.254	0.222	0.085 (0.147)	1.088	0.681
Daily pasta/bread/rice/potatoes. Yes vs. No	−0.468 (0.493)	0.626	0.518	−0.651 (0.413)	0.521	0.351	0.012 (0.091)	1.012	0.893
If yes. daily servings: 3–4 vs. 1–2	−3.255 (1.878)	0.039	0.129	−4.427 (1.667)	0.012	0.027	0.489 (0.346)	1.631	0.161
If no. weekly servings: 3–4 vs. 1–2	1.124 (1.026)	3.077	0.712	−0.36 (0.965)	0.697	0.712	0.127 (0.214)	1.135	0.712
If no. weekly servings: >4 vs. 1-2	1.817 (1.114)	6.15	0.174	−2.44 (1.184)	0.087	0.15	0.193 (0.251)	1.213	0.452
Daily Fruits and vegetables: *Yes vs. No*	−0.419 (0.605)	0.658	0.609	0.988 (0.475)	2.685	0.12	0.055 (0.106)	1.056	0.609
If yes. daily servings: 3–4 vs. 1–2	0.227 (0.487)	1.255	0.642	−0.614 (0.412)	0.541	0.42	−0.077 (0.082)	0.926	0.528
If yes. daily servings: >4 vs. 1–2	−1.977 (1.053)	0.139	0.096	−1.229 (1.227)	0.292	0.319	0.506 (0.199)	1.658	0.039
Weekly meat: at least once a day vs. at least once a week	−0.107 (0.637)	0.899	0.867	0.664 (0.545)	1.943	0.675	−0.065 (0.107)	0.937	0.816
Weekly meat: less than once a week vs. at least once a week	−0.141 (0.569)	0.868	0.804	0.321 (0.495)	1.379	0.777	−0.139 (0.098)	0.87	0.483
Weekly fish: *3–4 vs. 1–2*	0.383 (0.589)	1.467	0.517	−2.498 (0.471)	0.082	<0.001	−0.069 (0.098)	0.933	0.517
Weekly fish: *>4 vs. 1–2*	−2.492 (1.869)	0.083	0.277	−1.295 (1.698)	0.274	0.447	−0.553 (0.349)	0.575	0.277
Weekly fish: every 10–15 days vs. 1–2	−1.003 (0.525)	0.367	0.151	−0.686 (0.45)	0.503	0.151	−0.15 (0.103)	0.861	0.151
Weekly fish: never vs. 1–2	−0.798 (0.636)	0.45	0.212	−0.735 (0.55)	0.48	0.212	0.149 (0.103)	1.161	0.212
Weekly eggs: less than once a week vs. at least once a week	0.419 (0.504)	1.52	0.597	−0.237 (0.447)	0.789	0.597	0.056 (0.086)	1.058	0.597
Weekly cheese: *3–4 vs. 1–2*	1.316 (0.462)	3.729	0.015	−0.421 (0.417)	0.656	0.471	−0.065 (0.09)	0.937	0.471
Weekly cheese: *>4 vs. 1–2*	1.707 (0.508)	5.514	0.003	−0.7 (0.461)	0.497	0.198	−0.086 (0.101)	0.917	0.394
Weekly cheese: *every 10–15 days vs. 1–2*	0.461 (0.848)	1.585	0.971	−0.026 (0.705)	0.975	0.971	0.035 (0.146)	1.035	0.971
Weekly cheese: *never vs. 1–2*	−0.509 (0.693)	0.601	0.696	−2.164 (0.607)	0.115	0.003	0.023 (0.131)	1.023	0.863
Weekly cured meat: *3–4 vs. 1–2*	−0.058 (0.687)	0.943	0.932	−2.451 (0.543)	0.086	<0.001	−0.113 (0.129)	0.893	0.573
Weekly cured meat: *>4 vs. 1–2*	−0.142 (0.818)	0.868	0.863	−1.349 (0.642)	0.259	0.114	0.032 (0.135)	1.032	0.863
Weekly cured meat: *every 10–15 days vs. 1–2*	0.261 (0.505)	1.298	0.607	−0.925 (0.41)	0.396	0.078	−0.105 (0.091)	0.901	0.381
Weekly cured meat: *never vs. 1–2*	0.29 (0.575)	1.337	0.615	−1.31 (0.486)	0.27	0.024	0.123 (0.097)	1.131	0.312
Weekly legumes: *3–4 vs. 1–2*	−1.586 (0.524)	0.205	0.009	0.199 (0.454)	1.22	0.934	0.008 (0.099)	1.008	0.934
Weekly legumes: *>4 vs. 1–2*	−2.396 (1.313)	0.091	0.213	−1.121 (1.043)	0.326	0.428	−0.065 (0.212)	0.937	0.759
Weekly legumes: *every 10–15 days vs. 1–2*	−0.361 (0.492)	0.697	0.465	0.614 (0.462)	1.848	0.465	0.073 (0.097)	1.076	0.465
Weekly legumes: *never vs. 1–2*	0.759 (0.66)	2.136	0.759	−0.076 (0.545)	0.927	0.936	−0.009 (0.115)	0.991	0.936
Weekly cakes: *3–4 vs. 1–2*	0.404 (0.756)	1.498	0.647	−0.451 (0.649)	0.637	0.647	−0.059 (0.127)	0.943	0.647
Weekly cakes: *every day vs. 1–2*	0.118 (0.532)	1.125	0.825	−0.256 (0.469)	0.774	0.825	0.109 (0.093)	1.115	0.726
Weekly cakes: *2 per day vs. 1–2*	−0.684 (0.696)	0.504	0.492	−0.94 (0.649)	0.391	0.453	0.062 (0.115)	1.064	0.591
Weekly cakes: *every 10–12 days vs. 1–2*	−0.433 (0.653)	0.649	0.764	−0.127 (0.609)	0.881	0.835	−0.117 (0.127)	0.89	0.764
Weekly cakes: *never vs. 1–2*	−0.913 (0.795)	0.401	0.381	0.232 (0.744)	1.262	0.755	−0.224 (0.148)	0.799	0.381
Weekly French fries: *3–4 vs. 1–2*	−7.757 (1.829)	0.0004	<0.001	−3.289 (1.814)	0.037	0.108	−0.271 (0.374)	0.762	0.471
Weekly French fries: *every 10–15 days vs. 1–2*	−3.085 (0.682)	0.046	<0.001	1.34 (0.608)	3.82	0.045	0.056 (0.132)	1.058	0.672
Weekly French fries: never vs. 1–2	−0.673 (0.619)	0.51	0.419	0.996 (0.544)	2.708	0.21	0.041 (0.122)	1.042	0.736
Weekly fast food: every 10–15 days vs. 1–2	−2.86 (1.705)	0.057	0.288	−0.272 (1.491)	0.762	0.856	0.34 (0.309)	1.404	0.411
Weekly fast food: never vs. 1–2	−1.546 (1.336)	0.213	0.294	3.395 (1.23)	29.8	0.021	0.269 (0.255)	1.308	0.294
Weekly pizzeria: every 10–15 days vs. 1–2	−0.075 (0.526)	0.928	0.887	−0.129 (0.451)	0.879	0.887	−0.139 (0.091)	0.87	0.396
Weekly pizzeria: never vs. 1–2	−0.288 (0.496)	0.75	0.844	0.05 (0.437)	1.051	0.909	−0.247 (0.09)	0.781	0.021
Wine Yes vs. No	−0.297 (0.392)	0.743	0.675	−0.102 (0.343)	0.903	0.766	−0.061 (0.072)	0.941	0.675
Weekly wine: 3–4 vs. 1–2	1.368 (0.907)	3.926	0.137	−1.511 (0.781)	0.221	0.129	−0.362 (0.207)	0.696	0.129
weekly wine: every 10–15 days vs. 1–2	0.71 (0.773)	2.035	0.543	1.106 (0.682)	3.022	0.33	−0.086 (0.15)	0.918	0.57
weekly wine: never vs. 1–2	0.83 (0.656)	2.293	0.211	1.55 (0.601)	4.711	0.036	−0.259 (0.135)	0.772	0.09
Beer. Yes vs. No	0.251 (0.429)	1.286	0.779	−1.165 (0.352)	0.312	0.003	−0.022 (0.078)	0.978	0.779
Weekly beer: 3–4 vs. 1–2	0.664 (1.996)	1.942	0.742	−2.33 (1.07)	0.097	0.093	−0.48 (0.246)	0.619	0.093
weekly beer: every 10–15 days vs. 1–2	0.716 (0.798)	2046	0.376	2.108 (0.519)	8.232	<0.001	−0.144 (0.131)	0.866	0.376
weekly beer: never vs. 1–2	−3.584 (1.245)	0.028	0.021	−1.596 (0.8)	0.203	0.081	0.006 (0.187)	1.006	0.973
Other aperitifs and	−0.358 (0.477)	0.699	0.454	−1.656 (0.395)	0.191	<0.001	0.107 (0.094)	1.113	0.386
alcoholic drinks. Yes vs. No									
Weekly other aperitifs and	0.347 (1.759)	1.415	0.845	−2.221 (1.583)	0.109	0.525	−0.214 (0.296)	0.808	0.726
alcoholic drinks: 3–4 vs. 1–2									
Weekly aperitifs and	−2.024 (0.795)	0.132	0.057	0.769 (0.662)	2.158	0.388	−0.107 (0.148)	0.898	0.482
alcoholic drinks: every 10–15 days vs. 1–2									
Weekly aperitifs and	−1.755 (1.759)	0.173	0.495	−2.093 (1.165)	0.123	0.261	0.02 (0.224)	1.02	0.931
alcoholic drinks: never vs. 1–2									
Spirits Yes vs. No	0.491 (0.964)	1.634	0.612	1.121 (0.806)	3.067	0.25	0.303 (0.18)	1.353	0.25
24h Dietary Recall									
Number of meals in the 24h recall	−0.069 (0.119)	0.934	0.793	0.028 (0.107)	1.028	0.793	0.013 (0.021)	1.013	0.793
Alcoholic beverages	0.41 (0.297)	1.507	0.507	−0.26 (0.272)	0.771	0.512	−0.027 (0.057)	0.973	0.63
Non−alcoholic beverages	0.032 (0.164)	1.032	0.995	0.001 (0.149)	1.001	0.995	0.041 (0.029)	1.042	0.471
Condiments and sauces	−0.175 (0.318)	0.84	0.584	0.66 (0.281)	1.934	0.063	0.052 (0.064)	1.053	0.584
Fats and oils	−0.072 (0.262)	0.931	0.784	−0.421 (0.226)	0.657	0.195	0.052 (0.048)	1.053	0.43
Fruit	0.238 (0.205)	1.268	0.372	0.409 (0.176)	1.505	0.066	0.026 (0.036)	1.026	0.471
Grain products	−0.038 (0.165)	0.962	0.817	0.039 (0.147)	1.04	0.817	0.007 (0.03)	1.007	0.817
Milk and dairy	0.135 (0.239)	1.144	0.861	0.028 (0.193)	1.029	0.884	0.035 (0.042)	1.036	0.861
Mixed dishes	0.526 (0.325)	1.692	0.327	0.291 (0.274)	1.338	0.436	0.014 (0.054)	1.014	0.791
Potatoes: Yes vs. No	0.14 (0.868)	1.15	0.872	−0.314 (0.651)	0.731	0.872	−0.383 (0.136)	0.682	0.018
Protein food	0.112 (0.251)	1.119	0.808	−0.222 (0.233)	0.801	0.808	0.012 (0.048)	1.012	0.808
Snacks and sweets	0.204 (0.225)	1.226	0.366	0.265 (0.183)	1.304	0.225	0.079 (0.041)	1.082	0.171
Sugars	0.095 (0.192)	1.1	0.709	0.297 (0.168)	1.346	0.237	0.013 (0.035)	1.013	0.709
Vegetables	0.003 (0.236)	1.003	0.991	−0.508 (0.195)	0.602	0.03	−0.001 (0.041)	0.999	0.991
Water	−0.263 (0.187)	0.769	0.243	−0.277 (0.157)	0.758	0.243	−0.03 (0.034)	0.971	0.376

**Table 4:** Multivariable analysis for premature ventricular complexes, supraventricular premature complexes, and heart rate variability. Estimates are changes in PVCs for a unit increase of each of the predictors, Exp (Estimate) represents E[(Y+1); X]/ E [Y; X] indicating the relative variation in the expected number of arrhythmic events consequently to a unit increase of each considered X variable.

	Estimate	Exp (Estimate)	Standard Error	*p*-Value
Heart Rate Variability				
Age	−0.0005	0.9994	0.002	0.827
Sex: Female vs. Male	0.022	1.023	0.082	0.780
Sleep hours	0.058	1.059	0.027	0.034
BMI	−0.017	0.983	0.009	0.083
Fruit (24 h recall)	0.087	1.091	0.042	0.044
Premature Ventricular Complexes				
Age	0.06	1.062	0.011	0.005
Sex: Female vs. Male	−1.489	0.226	0.743	0.055
Fruit (24 h recall)	−0.893	0.409	0.375	0.024
Grain-based products (24 h recall)	1.24677	3.479	0.352	0.001
Snacks and sweets	0.68307	1.979	0.352	0.063
Sugars	0.86821	2.383	0.327	0.013
Mass of body fat	0.232	1.26	0.052	<0.001
Supraventricular Premature Complexes				
Age	0.049	1.050	1.039	<0.001
Sex: Female vs. Male	−0.909	0.403	0.308	0.004
Sleep hours	−0.299	0.741	0.108	0.006
Cardiovascular comorbidities: Yes vs. No	1.893	6.638	0.332	<0.001
Condiments and sauces	0.661	1.937	0.254	0.01
Protein food	−0.764	0.466	0.215	<0.001

## Supplementary Materials

The following are available online at https://www.mdpi.com/2077-0383/9/4/1121/s1, Figure S1: Distribution of Premature Ventricular Complexes, Figure S2: Distribution of Supraventricular Premature Complexes, Figure S3: Distribution of Heart Rate Variability, Table S1: Descriptive characteristic of subjects according with presence/absence of SVPCs.

## References

[1] Balakumar P., Maung-U K., Jagadeesh G. (2016). Prevalence and prevention of cardiovascular disease and diabetes mellitus. Pharmacol. Res..

[2] Payne R.A. (2012). Cardiovascular risk. Br. J. Clin. Pharmacol..

[3] Pais P., Kamath D.Y., Sigamani A., Xavier D. (2015). Prevention of Cardiovascular Disease: The Polypill Concept. Pathophysiology and Pharmacotherapy of Cardiovascular Disease.

[4] Maheshwari A., Norby F.L., Soliman E.Z., Adabag S., Whitsel E.A., Alonso A., Chen L.Y. (2016). Low heart rate variability in a 2-minute electrocardiogram recording is associated with an increased risk of sudden cardiac death in the general population: The atherosclerosis risk in communities study. PLoS ONE.

[5] Huikuri H.V., Stein P.K. (2013). Heart rate variability in risk stratification of cardiac patients. Prog. Cardiovasc. Dis..

[6] Sacha J. (2014). Interaction between heart rate and heart rate variability. Ann. Noninvasive Electrocardiol..

[7] Jain A., Aggarwal K., Zhang P. (2015). Omega-3 fatty acids and cardiovascular disease. Eur. Rev. Med. Pharm. Sci..

[8] Singer P., Wirth M. (2004). Can n-3 PUFA reduce cardiac arrhythmias? Results of a clinical trial. ProstaglandinsLeukot. Essent. Fat. Acids.

[9] Gogolashvili N., Litvinenko M., Pochikaeva T., Vavitova E., Polikarpov L., Novgorodtseva N. (2010). Possibilities of a preparation omega-3 polyunsaturated fatty acids in the treatment of patients with ventricular arrhythmias and myocardial infarction. Kardiologiia.

[10] Christensen J.H. (2011). Omega-3 polyunsaturated fatty acids and heart rate variability. Front. Physiol..

[11] Christensen J.H. (2003). n-3 fatty acids and the risk of sudden cardiac death. Emphasis on heart rate variability. Dan. Med. Bull..

[12] Karpyak V.M., Romanowicz M., Schmidt J.E., Lewis K.A., Bostwick J.M. (2014). Characteristics of Heart Rate Variability in Alcohol-Dependent Subjects and Nondependent Chronic Alcohol Users. Alcohol. Clin. Exp. Res..

[13] Voskoboinik A., Prabhu S., Ling L.-H., Kalman J.M., Kistler P.M. (2016). Alcohol and Atrial Fibrillation: A Sobering Review. J. Am. Coll. Cardiol..

[14] Nakajima K., Suwa K., Oda E. (2014). Atrial fibrillation may be prevalent in individuals who report late-night dinner eating and concomitant breakfast skipping, a complex abnormal eating behavior around sleep. Int. J. Cardiol..

[15] Fatisson J., Oswald V., Lalonde F. (2016). Influence diagram of physiological and environmental factors affecting heart rate variability: An extended literature overview. Heart Int..

[16] Plourde B., Sarrazin J.-F., Nault I., Poirier P. (2014). Sudden cardiac death and obesity. Expert Rev. Cardiovasc. Ther..

[17] Yadav R.L., Yadav P.K., Yadav L.K., Agrawal K., Sah S.K., Islam M.N. (2017). Association between obesity and heart rate variability indices: An intuition toward cardiac autonomic alteration–a risk of CVD. DiabetesMetab. Syndr. Obes. Targets Ther..

[18] Balestroni G., Bertolotti G. (2015). EuroQol-5D (EQ-5D): An instrument for measuring quality of life. Monaldi Arch. Chest Dis..

[19] Turconi G., Bazzano R., Roggi C., Cena H. (2010). Reliability and relative validity of a quantitative food-frequency questionnaire for use among adults in Italian population. Int. J. Food Sci. Nutr..

[20] U.S. Department of Agriculture, Agricultural Research Service (2016). What We Eat in America Food Categories 2013–2014. www.ars.usda.gov/nea/bhnrc/fsrg.

[21] Akhandaf Y., Van Klaveren J., De Henauw S., Van Donkersgoed G., Van Gorcum T., Papadopoulos A., Sirot V., Kennedy M., Pinchen H., Ruprich J. (2015). Exposure assessment within a Total Diet Study: A comparison of the use of the pan-European classification system FoodEx-1 with national food classification systems. Food Chem. Toxicol..

[22] Rigby R.A., Stasinopoulos D.M. (2005). Generalized additive models for location, scale and shape. J. R. Stat. Soc. Ser. C (Appl. Stat. ).

[23] Stasinopoulos D.M., Rigby R.A. (2007). Generalized additive models for location scale and shape (GAMLSS) in R. J. Stat. Softw..

[24] Agogo G.O. (2017). A zero-augmented generalized gamma regression calibration to adjust for covariate measurement error: A case of an episodically consumed dietary intake. Biom. J..

[25] Team R. (2013). R development core team. Ra Lang. Env. Stat. Comput.

[26] Chow G.V., Marine J.E., Fleg J.L. (2012). Epidemiology of arrhythmias and conduction disorders in older adults. Clin. Geriatr. Med..

[27] Cameron A., Magliano D., Söderberg S. (2013). A systematic review of the impact of including both waist and hip circumference in risk models for cardiovascular diseases, diabetes and mortality. Obes. Rev..

[28] Pathak R.K., Mahajan R., Lau D.H., Sanders P. (2015). The implications of obesity for cardiac arrhythmia mechanisms and management. Can. J. Cardiol..

[29] Samanta R., Pouliopoulos J., Thiagalingam A., Kovoor P. (2016). Role of adipose tissue in the pathogenesis of cardiac arrhythmias. Heart Rhythm.

[30] Park S.K., Tucker K.L., O'neill M.S., Sparrow D., Vokonas P.S., Hu H., Schwartz J. (2009). Fruit, vegetable, and fish consumption and heart rate variability: The Veterans Administration Normative Aging Study. Am. J. Clin. Nutr..

[31] Silvetti M.S., Drago F., Ragonese P. (2001). Heart rate variability in healthy children and adolescents is partially related to age and gender. Int. J. Cardiol..

[32] Van Dam R., Seidell J. (2007). Carbohydrate intake and obesity. Eur. J. Clin. Nutr..

[33] Reiffel J.A., McDonald A. (2006). Antiarrhythmic effects of omega-3 fatty acids. Am. J. Cardiol..

[34] Stein P.K., Pu Y. (2012). Heart rate variability, sleep and sleep disorders. Sleep Med. Rev..

[35] Miner S.E.S., Pahal D., Nichols L., Darwood A., Nield L.E., Wulffhart Z. (2016). Sleep Disruption is Associated with Increased Ventricular Ectopy and Cardiac Arrest in Hospitalized Adults. Sleep.

