# Supplementary material for: Dietary and Lifestyle Patterns are Associated with Heart Rate Variability
Source: J Clin Med. 2020 Apr 14;9(4):1121. doi: 10.3390/jcm9041121 (PMC7230664; doi:10.3390/jcm9041121)
Supplement: Supplementary file 1 [file jcm-09-01121-s001.pdf]

Journal of
Clinical Medicine

MDPI

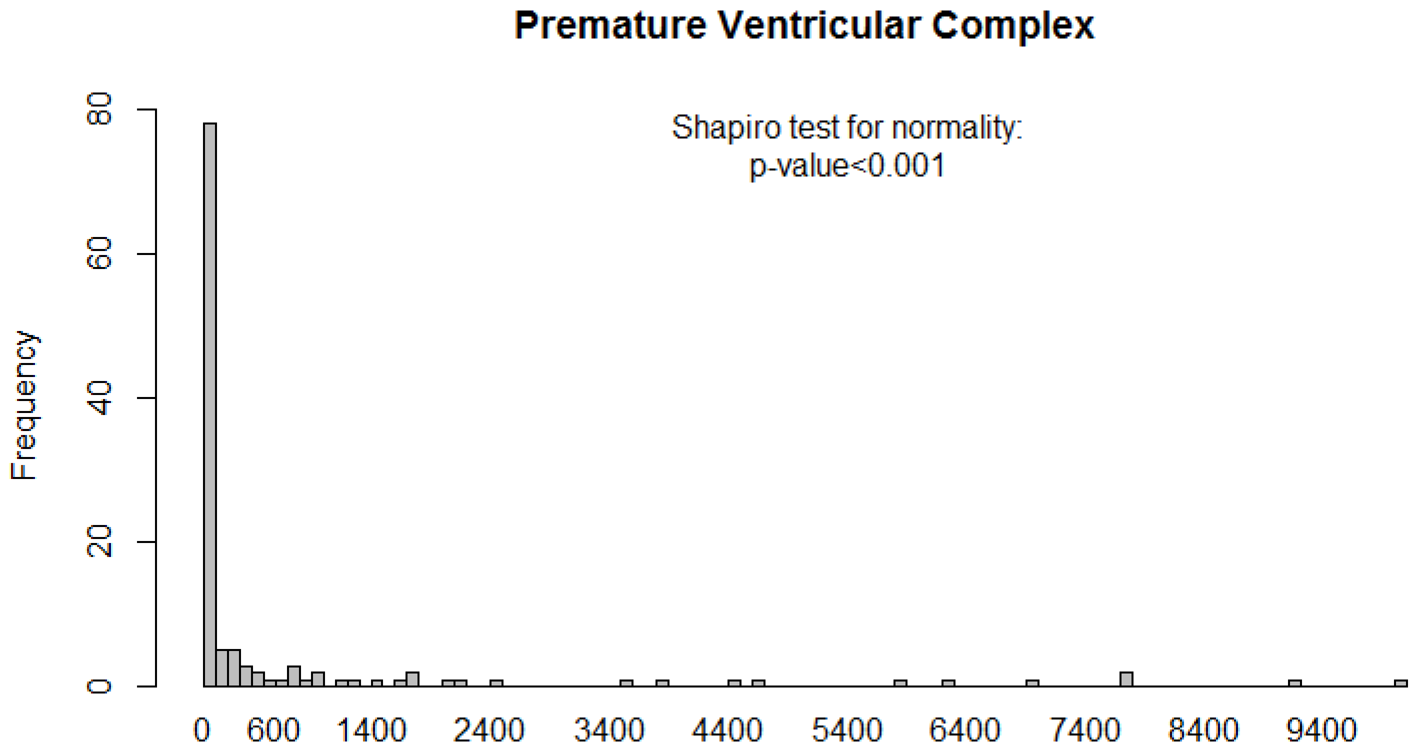

Figure S1. Distribution of Premature Ventricular Complexes. The x-axis reports the number of Premature Ventricular Complexes.

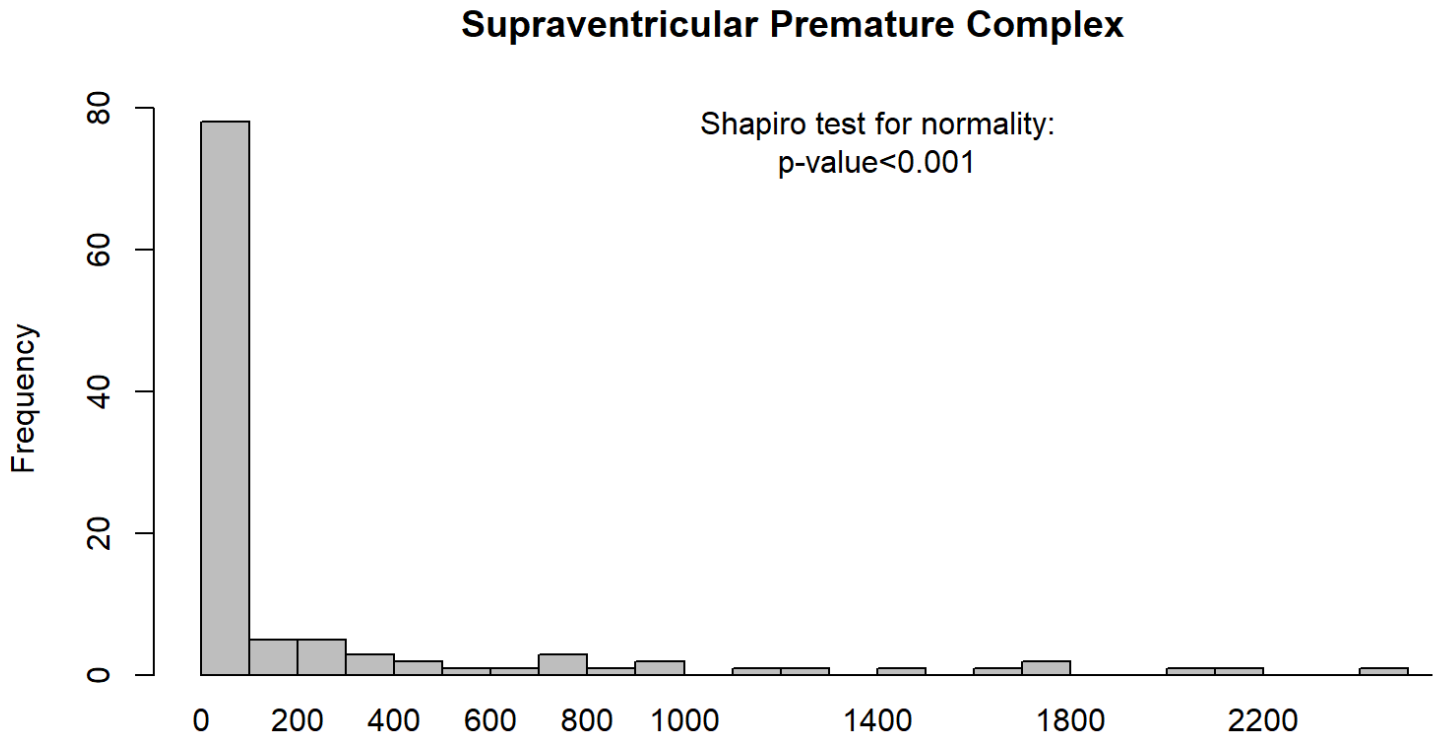

Figure S2. Distribution of Supraventricular Premature Complexes. The x-axis reports the number of Supraventricular Premature Complexes.

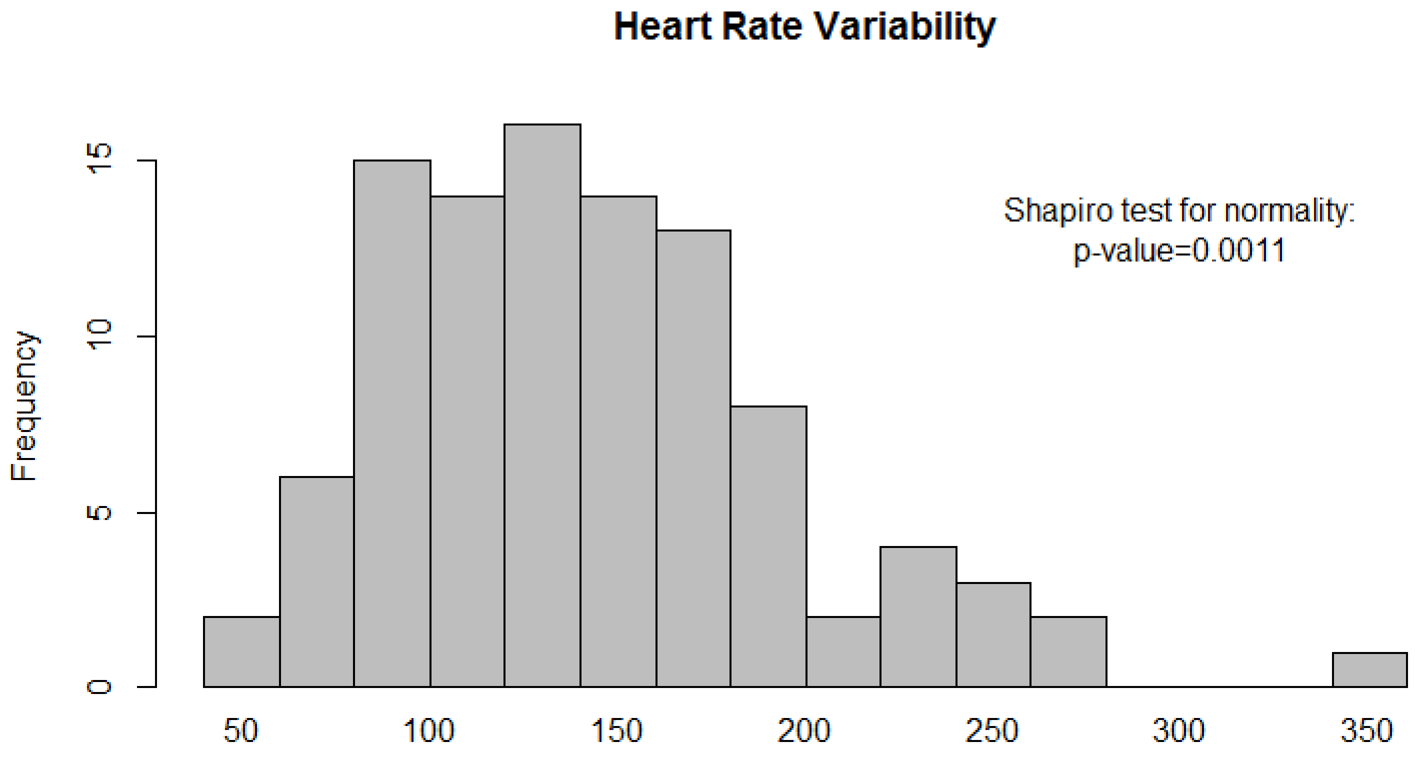

Figure S3. Distribution of Heart Rate Variability. The x-axis reports the Heart Rate Variability (standard deviation of the N-N (SDNN) intervals in ms)

Table 1. Descriptive characteristic of subjects according with presence/absence of Supraventricular Premature Complexes

	Supraventricular Premature Complexes: No (*n*=12)	Supraventricular Premature Complexes: Yes (*n*=109)	Combined (*n*=121)	*p*-Value
**Socio-demographic characteristics and lifestyle**				
Age	26.25/45.50/73.75	49.00/66.00/75.00	47.00/66.00/75.00	0.148
Gender: Male	50% (6)	43% (47)	44% (53)	0.648
Female	50% (6)	57% (62)	56% (68)	
Educational level: Low	25% (3)	48% (52)	45% (55)	0.314
Medium	42% (5)	31% (34)	32% (39)	
High	33% (4)	21% (23)	22% (27)	
Employment: No	67% (8)	64% (70)	64% (78)	0.867
Yes	33% (4)	36% (39)	36% (43)	
Cardiovascular comorbidities: No	33% (4)	43% (47)	42% (51)	0.515
Yes	67% (8)	57% (62)	58% (70)	
Concomitant medications. Ace inhibitors: Yes	25% (3)	39% (42)	37% (45)	0.441
Diuretics: Yes	42% (5)	23% (25)	25% (30)	
Potassium supplements: Yes	0% (0)	2% (2)	2% (2)	
Flecainide: Yes	0% (0)	6% (6)	5% (6)	
Class I antiarrhythmic agents: Yes	0% (0)	1% (1)	1% (1)	
Class III antiarrhythmic agents: Yes	0% (0)	3% (3)	2% (3)	
Digoxin: Yes	0% (0)	2% (2)	2% (2)	
Nitrates: Yes	0% (0)	3% (3)	2% (3)	
Calcium channel blockers: Yes	25% (3)	14% (15)	15% (18)	
Beta blockers: Yes	25% (3)	36% (39)	35% (42)	
Vasodilators: Yes	0% (0)	1% (1)	1% (1)	
Platelets aggregation inhibitors: Yes	17% (2)	32% (35)	31% (37)	
Anticoagulants: Yes	33% (4)	11% (12)	13% (16)	
Cholesterol lowering medications: Yes	42% (5)	28% (30)	29% (35)	
Insulin: Yes	0% (0)	4% (4)	3% (4)	
Oral hypoglycaemic agents: Yes	8% (1)	8% (9)	8% (10)	
Smoking habit: No	58% (7)	59% (64)	59% (71)	0.921
Past smoker	25% (3)	28% (31)	28% (34)	

Current smoker	17% (2)	13% (14)	13% (16)	
Cigarettes number (per day)	5.50/7.00/8.50	5.00/9.00/20.00	4.75/9.00/20.00	0.542
Physical activity: No	42% (5)	61% (66)	59% (71)	0.207
Yes	58% (7)	39% (43)	41% (50)	
Physical activity, *number of weekly training session*	3.0/3.0/5.5	2.0/3.0/3.5	2.0/3.0/4.0	0.056
Sleep Hours	6.0/6.5/8.0	5.0/6.5/7.0	5.0/6.5/7.0	0.248
EQ5D VAS	60.00/70.00/82.00	51.00/70.00/80.00	57.75/70.00/80.00	0.979
**Anthropometrics and BIA**				
Lean Body Mass	48.95/50.80/55.85	43.30/45.95/52.92	43.85/46.20/52.92	0.155
Total Body Water	35.27/36.60/40.22	31.15/33.05/38.10	31.57/33.25/38.10	0.155
Extra cellular Water	14.20/14.55/16.30	12.50/13.45/15.52	12.57/13.75/15.52	0.132
Mass of Body Fat	9.45/11.85/17.60	16.05/20.25/22.37	14.85/19.75/22.37	0.195
Percent of Body Fat	15.10/18.80/25.37	23.05/30.10/33.85	21.60/29.65/33.85	0.195
BMI	21.40/23.28/27.01	22.37/24.77/27.67	22.28/24.66/27.67	0.537
Skinfold thickness	8.87/17.48/28.25	11.75/18.30/23.66	11.57/18.30/23.91	1
Waist	81.37/ 94.50/106.25	86.00/ 95.00/103.00	86.00/ 95.00/103.00	0.913
Hip	94.75/102.00/110.50	96.50/102.00/107.00	96.00/102.00/107.50	0.847
**Food Frequency Questionnaire**				
Score of eating habits section	42.0/46.0/48.5	42.0/45.0/49.0	42.0/45.0/49.0	0.779
Daily milk/yogurth: *At least once a week*	40% (2)	30% (9)	31% (11)	0.656
Less than once a week	60% (3)	70% (21)	69% (24)	
If yes, how many? 1–2	100% (7)	95% (75)	95% (82)	0.542
3–4	0% (0)	5% (4)	5% (4)	
If no, weekly milk/yogurth: 1–2	20% (1)	3% (1)	6% (2)	0.418
3–4	20% (1)	27% (8)	26% (9)	
>4	0% (0)	13% (4)	11% (4)	
10–15 days	60% (3)	57% (17)	57% (20)	
Daily grains: No	17% (2)	21% (23)	21% (25)	0.719
Yes	83% (10)	79% (86)	79% (96)	
If yes, how many? 1–2	100% (10)	99% (85)	99% (95)	0.732
3–4	0% (0)	1% (1)	1% (1)	
If no, weekly grains: 1–2	0% (0)	25% (6)	22% (6)	0.603

3–4	67% (2)	54% (13)	56% (15)	
>4	33% (1)	21% (5)	22% (6)	
Daily fruits and vegetables: No	25% (3)	15% (16)	16% (19)	0.351
Yes	75% (9)	85% (93)	84% (102)	
If yes, how many? 1–2	78% (7)	73% (68)	74% (75)	0.233
3–4	11% (1)	25% (23)	24% (24)	
>4	11% (1)	2% (2)	3% (3)	
Weekly servings of meat: at least once a week	58% (7)	75% (82)	74% (89)	0.441
At least once a day	17% (2)	11% (12)	12% (14)	
Less than once a week	25% (3)	14% (15)	15% (18)	
Weekly servings of fish: 1–2	42% (5)	58% (63)	56% (68)	0.228
3–4	8% (1)	15% (16)	14% (17)	
> 4	0% (0)	1% (1)	1% (1)	
every 10–15 days	17% (2)	17% (18)	17% (20)	
never	33% (4)	10% (11)	12% (15)	
Weekly servings of eggs: at least once a week	58% (7)	83% (90)	80% (97)	0.046
Less than once a week	42% (5)	17% (19)	20% (24)	
Weekly servings of cheese: 1–2	58% (7)	39% (42)	40% (49)	0.625
3–4	8% (1)	27% (29)	25% (30)	
>4	17% (2)	19% (21)	19% (23)	
every 10–15 days	8% (1)	6% (7)	7% (8)	
never	8% (1)	9% (10)	9% (11)	
Weekly servings of cured meat: 1–2	25% (3)	43% (47)	41% (50)	0.45
3–4	17% (2)	11% (12)	12% (14)	
>4	8% (1)	7% (8)	7% (9)	
every 10–15 days	17% (2)	24% (26)	23% (28)	
never	33% (4)	15% (16)	17% (20)	
Weekly servings of legumes: 1–2	42% (5)	48% (52)	47% (57)	0.841
3–4	25% (3)	19% (21)	20% (24)	
>4	8% (1)	3% (3)	3% (4)	
every 10–15 days	17% (2)	18% (20)	18% (22)	
never	8% (1)	12% (13)	12% (14)	

Weekly servings of cakes: 1–2	0% (0)	28% (27)	26% (27)	0.244
3–4	10% (1)	11% (10)	10% (11)	
1 per day	60% (6)	31% (29)	33% (35)	
2 per day	20% (2)	11% (10)	11% (12)	
every 10–15 days	10% (1)	13% (12)	12% (13)	
never	0% (0)	7% (7)	7% (7)	
Weekly servings of french fries: 1–2	0% (0)	11% (12)	10% (12)	0.608
3–4	0% (0)	1% (1)	1% (1)	
every 10–15 days	33% (4)	24% (26)	25% (30)	
never	67% (8)	64% (70)	64% (78)	
How often do you eat at fast-food per week? (weekly) 1–2	0% (0)	2% (2)	2% (2)	0.132
every 10–15 days	17% (2)	4% (4)	5% (6)	
never	83% (10)	94% (103)	93% (113)	
How often do you eat at pizzeria? (weekly) 1–2	36% (4)	24% (26)	25% (30)	0.317
3–4	45% (5)	35% (38)	36% (43)	
every 10–15 days	18% (2)	41% (45)	39% (47)	
Do you drink wine: No	75% (9)	43% (47)	46% (56)	0.036
Yes	25% (3)	57% (62)	54% (65)	
If yes, how many? (weekly) 1–2	0% (0)	16% (10)	15% (10)	0.751
3–4	0% (0)	13% (8)	12% (8)	
every 10–15 days	33% (1)	23% (14)	23% (15)	
every day	67% (2)	48% (30)	49% (32)	
Do you drink beer? No	83% (10)	67% (73)	69% (83)	0.246
Yes	17% (2)	33% (36)	31% (38)	
If yes, how many? (weekly): 1–2	50% (1)	42% (15)	42% (16)	0.941
3–4	0% (0)	6% (2)	5% (2)	
every 10–15 days	50% (1)	42% (15)	42% (16)	
every day	0% (0)	11% (4)	11% (4)	
Do you drink other alcholic beverages? No	91% (10)	78% (84)	79% (94)	0.308
Yes	9% (1)	22% (24)	21% (25)	
If yes, how many? (weekly): 1–2	0% (0)	46% (11)	44% (11)	0.723
3–4	0% (0)	4% (1)	4% (1)	

every 10–15 days	100% (1)	42% (10)	44% (11)	
every day	0% (0)	8% (2)	8% (2)	
Do you drink spirits? No	100% (12)	95% (104)	96% (116)	0.449
Yes	0% (0)	5% (5)	4% (5)	
**24-h Dietary Recall**				
Number of meals in the 24-h recall	2.75/3.50/4.25	3.00/4.00/5.00	3.00/4.00/5.00	0.387
Alcholic beverages	0/0/0	0/0/1	0/0/0	0.228
Non-alcoholic beverages	0.00/0.50/1.25	1.00/1.00/2.00	0.00/1.00/2.00	0.058
Condiments and Sauces	0.00/0.00/0.25	0.00/1.00/1.00	0.00/0.00/1.00	0.085
Fats and Oils	0.0/0.5/1.0	0.0/1.0/1.0	0.0/1.0/1.0	0.227
Fruit	0.00/0.00/1.25	0.00/1.00/2.00	0.00/1.00/2.00	0.339
Grain Products	0.00/1.00/1.25	1.00/2.00/2.00	1.00/1.00/2.00	0.057
Milk and Dairy	0/0/1	0/1/2	0/1/2	0.111
Mixed Dishes	0.0/0.5/1.0	0.0/0.0/1.0	0.0/0.0/1.0	0.146
Potatoes : No	100% (12)	93% (101)	93% (113)	0.331
Yes	0% (0)	7% (8)	7% (8)	
Protein food	0/1/1	0/1/1	0/1/1	0.805
Snacks and Sweets	0.0/1.5/2.0	0.0/1.0/1.0	0.0/1.0/2.0	0.126
Sugars	0/0/1	0/1/2	0/1/2	0.042
Vegetables	0.00/1.00/1.25	0.00/1.00/2.00	0.00/1.00/2.00	0.443
Water	0/1/2	0/1/2	0/1/2	0.814
